# Kinetic analysis suggests that malathion at low environmental exposures is not toxic to humans

**DOI:** 10.1371/journal.pone.0335361

**Published:** 2025-10-24

**Authors:** Lawrence M. Schopfer, Ozden Tacal, Patrick Masson, Oksana Lockridge

**Affiliations:** 1 Eppley Institute, University of Nebraska Medical Center, Nebraska Medical Center, Omaha, Nebraska, United States of America; 2 Department of Biochemistry, School of Pharmacy, Hacettepe University, Ankara, Turkey; 3 Biochemical Neuropharmacology Laboratory, Kazan Federal University, Kazan, Russia; Al-Jouf University College of Medicine, SAUDI ARABIA

## Abstract

Malathion has the reputation of being a safe pesticide. There are no reported cases of cholinergic toxicity in people exposed to low environmental doses of malathion. Our goal was to explain the safety of malathion in terms of the mechanism of malathion detoxication. The structure of malathion includes a built-in safety feature, specifically two ethyl esters. The ethyl esters are decarboxylated by human esterases to negatively charged malathion which does not react with acetylcholinesterase. Acetylcholinesterase is the toxicologically relevant target for organophosphates such as malathion. A toxic form of malathion is produced by Cytochrome P450 enzymes which convert malathion to malaoxon. Malaoxon is toxic because it inhibits acetylcholinesterase. We used high pressure liquid chromatography on a Prodigy 5 µm ODS column to monitor the production of enzyme-catalyzed decarboxylation of the malathion ethyl esters. The products of malathion decarboxylation were identified by mass spectrometry using a Thermo RSLC Ultimate 3000 ultra-high pressure chromatography system with a Thermo Easy-Spray PepMap RSLC C18 separation column attached to an Orbitrap Fusion Lumos Tribrid mass spectrometer. Decarboxylation and enzyme inhibition were assayed with recombinant human acetylcholinesterase (rHuAChE), human butyrylcholinesterase (HuBChE), and recombinant human liver carboxylesterase (rHuCE1). A trace contaminant in 98.5% pure malathion was identified by mass spectrometry. Consistent with the fact that negatively charged compounds are not inhibitors of HuAChE, HuBChE, or HuCE1, we found that negatively charged, decarboxylated malathion did not inhibit the activity of rHuAChE, HuBChE, or rHuCE1. Carboxylesterase detoxified malathion 100,000-fold faster compared to rHuAChE and HuBChE. Low dose exposures to malathion are not directly toxic The toxic metabolite, malaoxon, is produced very slowly. By comparison, detoxified malathion acids are formed rapidly. In conclusion, our data suggest that the safety of low dose environmental exposures to malathion is explained by the fact that malathion is detoxified faster than it is activated to the toxic malaoxon. Our review of the literature finds no convincing evidence that low dose malathion exposure causes cancer.

## Introduction

Malathion, also called Karbofos, is one of the most common organophosphothioate pesticides currently in use. The Environmental Protection Agency states that there is “no human health risks of concern from the registered uses of malathion” [1]. However, over the past 20 years, an increasing number of papers have appeared suggesting that malathion poses health risks. In particular, it was pointed out that the presence of impurities in preparations may enhance toxicity of malathion [2]. These studies use malathion concentrations well in excess of the levels encountered in the environment due to pest control. Unfortunately, too many researchers do not read papers that are over 20 years old, making the perception that malathion is toxic more commonly believed. It is time that this growing body of misleading data be refuted by re-emphasizing the safety of malathion. In the current work, we have reinvestigated some of the properties of malathion to re-emphasize its safety, have addressed some of the work that has called that safety into question, and have provided direct evidence that apparent inhibition of HuBChE by malathion is due to isomalathion contamination.

Malathion is applied by spraying which makes dermal absorption the principal route of contamination for people. Contamination by breathing the pesticide would be expected to occur, but the amount of malathion that is deposited on exposed skin is 20–1700 times the amount reaching the respiratory tract [3]. In addition, absorption of malathion through skin is poor. Only 1–8% of the applied amount is absorbed [3–7]. In contrast, 95% of malathion administered orally is absorbed [6]. The standard method for estimating the level of absorbed malathion is to measure the malathion metabolic products, mono- and dicarboxylic acids, in the urine [8]. Combining the percent absorption with the measured level of metabolites in urine, exposure of workers to malathion in the field can be estimated. It is found that typical exposures are 0.0046–1.1 mg/kg/day [4,6,9]. It is interesting to note that the LD50 for malathion in humans is 858 mg/kg [10], a value 3 orders of magnitude higher than environmental exposure levels.

In addition to the absorption safe-guard, malathion is subject to chemical safe-guards. First, organophosphorus compounds are toxic because they inhibit acetylcholinesterase. Malathion does not react directly with acetylcholinesterase. In order to become toxic, malathion must be oxidized to malaoxon by cytochrome P450. The P450 isozyme CYP2C19 has the highest affinity and fastest Vmax for oxidative desulfuration of malathion. The apparent second order rate constant for human liver microsomal CYP2C19 (Vmax/Km) is 0.040 nmol/(min_mg protein_µM) [11]. Second, malathion is detoxified by carboxylesterase mediated decarboxylation that converts neutral malathion to negatively charged malathion monocarboxylic and dicarboxylic acids [12–14]. These negatively charged metabolites are poor inhibitors of acetylcholinesterase [13]. The apparent second order rate constant for decarboxylation by human liver microsomal carboxylesterase is 30 nmol/(min_mg protein_µM) [13]. This makes hydrolytic inactivation of malathion by carboxylesterase 750-fold faster than cytochrome P450 activation, strongly favoring inactivation over activation.

**Fig 1:**
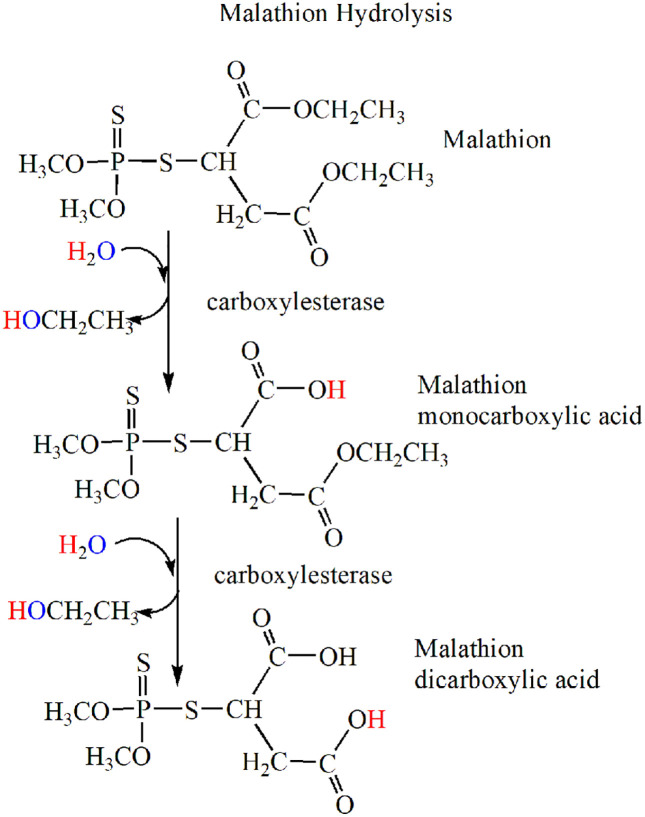
Steps in the hydrolysis of the ethyl esters of malathion to nontoxic acids. Carboxylesterase de-esterifies malathion in two-steps. The monocarboxylic acid and the dicarboxylic acid are nontoxic.

The relevant reactions in malathion ethyl-ester hydrolysis are shown in Fig 1.

In view of the low levels of malathion absorption and the preference for inactivation over activation it is not surprising that reports of adverse effects from environmental exposure to malathion are rare. The following information was taken from the EPA Review of Malathion Incident Reports [15]. The National Pesticide Telecommunications Network received 95 reports of adverse health effects from exposure to malathion between 1984 and 1991. The Poison Control Centers received 10,637 reports on malathion cases between 1985 and 1992. There were 679 occupational exposures, 6357 adult non-occupational exposures, and 3601 child exposures. Of the 28 organophosphate pesticides and carbamates included in the reports, malathion had the 6^^th^^ highest ratio of poisonings to applications. During widespread spraying of malathion in Los Angeles county between 1982–1995, 539 reports of malathion exposure were received from the general population [California Department of Food and Agriculture was operating between 1982–1990 and California Department of Pesticide Regulation was operating between 1991–1995]. There were 510 reports of malathion poisoning from farm workers and 330 reports from pesticide handlers between 1982 and 1989 [California Department of Food and Agriculture]. The majority of these incidents involved minor symptoms such as headache, eye irritation, skin rash, nausea, and upper respiratory irritation which in many cases may have been a reaction to the odor of the spray. There were no reports of cholinergic toxicity from malathion at low environmental doses.

More recently, effort has turned toward finding a connection between malathion and cancer. Overall, there appears to be little evidence for a connection between malathion and cancer. The Office of Chemical Safety and Pollution Prevention in the Environmental Protection Agency cautioned that positive reports of genotoxicity in the literature should be interpreted with care. They reported that there was “suggestive evidence of carcinogenicity [for malathion] but not sufficient to assess human carcinogenic potential” [16]. Epidemiologic studies on the likelihood of farmers getting cancer from exposure to malathion were often contradictory and at best indicated a low increase in probability. For example, data from the British Columbia Cancer Registry indicated a 1.3-fold increased likelihood for prostate cancer [17]; data from the Agricultural Health Study, Iowa and North Carolina cohort indicated no association between malathion and increased risk for melanoma, bladder cancer, kidney cancer, colorectal cancer or prostate cancer [18]; data from the Agricultural Health Study, Iowa and North Carolina cohort indicated that women whose husbands used malathion had a 1.5 increased likelihood for breast cancer [19,20]; data from the Canadian Provincial Cancer Registries indicated that farmers had a 1.8-fold increased likelihood for non-Hodgkin’s lymphoma [20]; another study using data from the Agricultural Health Study, Iowa and North Carolina cohort indicated that farmers had a 2.04-fold increased likelihood for thyroid cancer and a 0.64-fold decreased likelihood for non-Hodgkin’s lymphoma [21]. Alavanja & Bonner reviewed occupational pesticide exposure and links to cancer and concluded that “Most pesticides have not been found to be associated with cancer in epidemiologic studies.” and “In the United States and other developed countries farmers experience a lower mortality and cancer incidence rate compared to the general population” [22,23].

Studies on rats were undertaken to identify potential risks of malathion exposure for people. Investigators found that intraperitoneal injection of 100 or 150 mg/kg malathion once-a-day for 28 days caused DNA damage in the rat hippocampus [23], and that intraperitoneal injection of 170 mg/kg malathion twice-a-day for 5 days induced adenocarcinoma in the rat mammary gland [24]. Both studies identified potential cancer risks from malathion. However, the dosage given to the rats in these studies is more than 100-fold higher than that experienced by people during environmental exposure.

Additional studies with cultured cells were performed to identify cancer risks. The results were inconsistent and suffered from the use of malathion at levels well above those expected from environmental exposure. The following are examples of studies that made a connection between malathion and cancer only when malathion doses were higher than environmental exposure doses. An environmental exposure dose would be a single dose of 0.02 to 46 µM malathion. Culturing human lymphocytes in the presence of 50 µg/ml malathion (0.15 mM) resulted in the up-regulation of 659 genes and the down-regulation of 3729 genes. Of these 57 genes were associated with cancer [25]. Culturing human mammary cells in the presence of 50 µg/ml malathion caused changes in gene frequency [26]. Culturing human mononuclear cells in the presence of phytohemagglutinin and 200 µM malathion for 24 hours resulted in breaks in gene KMT2A, a gene that is associated with hematological malignancies [27]. Culturing human T-lymphocytes in the presence of 0.09–1.8 mM malathion resulted in no increase in mutational frequency [10]. Culturing human peripheral lymphocytes in the presence of 25, 75, or 200 µM malathion caused no DNA damage.

In summary, there is no convincing evidence for a correlation between environmental exposure to malathion and cancer or any toxic symptoms. Most of the cancer associations were seen at supra-environmental doses.

## Materials and methods

### Materials

Human plasma butyrylcholinesterase (HuBChE) was purified from Cohn fraction IV-4 by affinity chromatography on Hupresin [28]. Pure HuBChE consists of 4 identical subunits, each with a molecular weight of 85 kDa. The specific activity of the pure tetramer is 500 unit/mg [28]. Units of activity for HuBChE and rHuAChE are µmoles per min at pH 7, 25˚C, where the substrate for HuBChE is 1 mM butyrylthiocholine and for rHuAChE is 1 mM acetylthiocholine. The thiocholine reaction product is detected by the yellow color of the product (5-thio-2-nitrobenzoic acid) resulting from 5,5’ dithio-bis-2-nitrobenzoic acid reduction by thiocholine at 412 nm, E_412nm_ = 13,000 M^^-1cm-1^^ [29]. One unit of HuBChE activity corresponds to 2 µg HuBChE protein and 23.5x10^^-12^^ moles of active sites [28]. Pure recombinant human carboxylesterase (rHuCE1) was a kind gift from Dr. Philip Potter [30]. Units of activity for rHuCE1 are µmoles p-nitrophenyl acetate hydrolyzed per min at 25˚C, where the substrate is 5 µM p-nitrophenyl acetate, the buffer is 1% Triton X-100 in 20 mM TrisCl pH 7.5, and the yellow p-nitrophenol product is monitored at 400 nm, E_400nm_ = 9000 M^^-1cm-1^^. Pure human recombinant acetylcholinesterase (rHuAChE) without a Histidine tag was expressed in Chinese Hamster Ovary cells and purified by affinity chromatography on Procainamide-Sepharose [31]. Malathion (cat # N121346, 98.5% pure), isomalathion (cat# MET-12346AJ1, 88% pure), and malaoxon (cat # MET-12346C, 91.5% pure) were from Chem Services (West Chester, PA). Malathion alpha monocarboxylic acid (cat # M111015, > 95% pure) and malathion beta monocarboxylic acid (cat # M111020, 94% pure) were from Toronto Research Chemicals Inc (Ontario, Canada). Malathion dicarboxylic acid (cat # DRE-C14713000, Ehrenstorfer standard, 99.0% pure) was from VHG Labs (Manchester, NH). Butyrylthiocholine (cat # 20850, > 99% pure) was from Fluka/Honeywell Research Chemicals (Morris Plains, NJ). Acetylthiocholine (cat# 5751, > 98% pure), 5,5’dithio-bis-2-nitrobenzoic acid (cat # D8130), and pepsin (cat #6887, porcine gastric mucosa) were from Sigma-Aldrich (St Louis, MO). Trifluoroacetic acid (cat # 29004, sequencing grade) was from Beckman (Indianapolis, IN). Formic acid (cat # A117, 25% solution, Optima LC/MS) and acetonitrile (cat# 11700, synthesis grade) were from Fisher Scientific (Lenexa, KS). Pierce C-18 spin column (cat #89870) was from Thermo Scientific (Waltham, MA). Ultrafree 0.22 µm Durapore centrifugal filter units (UFC30GV0S) were from Merck Millipore (Burlington, MA).

### Inhibition of HuBChE by malathion measured in a Gilford spectrophotometer at 25˚C

A 260 µl reaction mixture containing 3.9 units (91.7 moles = 0.35 µM) HuBChE and 0.011 to 126 mM malathion in 0.1 M potassium phosphate pH 7.0, was incubated at room temperature. The enzyme activity was confirmed before the assay. At times between 1 and 250 minutes, a 20 µl aliquot of the reaction mixture was transferred to 2 ml of 0.1 M potassium phosphate buffer pH 7.0 containing 1 mM butyrylthiocholine and 0.5 mM 5,5’-dithio-bis-2-nitrobenzoic acid. The residual HuBChE activity was measured at 412 nm (the Ellman assay) [29]. The 100-fold dilution of the reaction mixture into buffer containing 1 mM butyrylthiocholine was sufficient to displace non-covalently bound malathion. Assays were repeated 3-times and average values were used.

### Liquid chromatography tandem Mass spectrometry of the malathion/HuBChE adduct

The following experiment was performed to distinguish between inhibition by malathion and inhibition by a contaminant in the malathion preparation. A 100 µl reaction mixture contained 34 units/ml HuBChE (0.8 µM) and 3.7 mM malathion in 50 mM ammonium bicarbonate pH 8. After incubation at room temperature for 17 hours in the dark, 52% of the HuBChE activity remained. Control samples without added malathion retained 100% activity.

Organophosphate irreversible inhibition is due to formation of a covalent adduct on the active site serine (S198) of HuBChE [32]. Digestion of HuBChE with pepsin according to the method of Fidder et al, yields short active site peptides suitable for mass spectrometry analysis [32].

The malathion-inhibited HuBChE solution was adjusted to pH 2 with 1 µl of 25% trifluoroacetic acid. A 2 mg/ml pepsin solution in 5% formic acid was prepared immediately before use. The HuBChE solution was incubated with 6 µg pepsin for 2 hours, at room temperature, in the dark. The digest was dried in a SpeedVac, redissolved in 2% acetonitrile plus 0.1% formic acid, and the peptides purified on a Pierce C-18 spin column (cat# 89870, Thermo Scientific, Rockford, IL) according to the manufacturer’s instructions. The C-18 purified peptides were dried and redissolved in 20 µl of 2% acetonitrile plus 0.1% formic acid to give 4 µM peptides.

Peptides were analyzed by liquid chromatography tandem mass spectrometry. Peptide separation was obtained on a Thermo RSLC Ultimate 3000 ultra-high pressure liquid chromatography system (Thermo Scientific) at 36°C. Solvent A was 0.1% formic acid in water, and solvent B was 0.1% formic acid in 80% acetonitrile. Peptides were loaded onto an Acclaim PepMap 100 RSLC Nanoviper C18 trap column 75 μm × 2 cm (Thermo scientific, cat# 164535) at a flow rate of 4 µl/min and washed with 100% solvent A for 10 min. Then, the peptides were transferred to a Thermo Easy-Spray PepMap RSLC C18 separation column 75 μm × 50 cm with 2 μm particles (Thermo Scientific, cat# ES903) and separated at a flow rate of 300 nL/min using a gradient of 9 − 25% solvent B in 27 min, 25 − 35% solvent B in 5 min, 35 − 99% solvent B in 4 min, hold at 99% solvent B for 4 min, from 99 to 9% solvent B in 4 min, hold at 9% solvent B for 16 min. Eluted peptides were sprayed directly into an Orbitrap Fusion Lumos Tribrid mass spectrometer (Thermo Scientific). Data were collected using data-dependent acquisition. A survey full scan MS (from 350 to 1800 m/z) was acquired in the Orbitrap in positive ion mode, with a resolution of 120,000. The AGC target (Automatic Gain Control for setting the ion population in the Orbitrap before collecting the mass spectrum) was set at 4x10^^5^^, and the ion filling time was set at 100 ms. The 25 most intense ions with charge state of 2 − 6 were isolated in quadrupole mode, in a 3 s cycle, and fragmented using high-energy collision-induced dissociation with 35% normalized collision energy. Fragment ions were detected in the Orbitrap with a mass resolution of 30,000 at 200 m/z. The AGC target for the mass spectral fragmentation spectrum was set at 5x10^^4^^, ion filling time was set at 60 ms, and dynamic exclusion set for 30 s after 1 time with a 10 ppm mass window. Data were reported in *.raw format.

Mass spectral data in *.raw format were converted to *.mgf with MSConvert (Source Forge/Proteome Wizard) [33]. Candidates for phosphylated HuBChE peptides were identified by Protein Prospector v 6.4.9 (prospector.ucsf.edu/prospector/mshome.htm) database searches using Batch-Tag Web. The search parameters were as follows: Database was User protein; User Protein Sequence was the FASTA file for HuBChE (accession number P06276); Digest was no enzyme; Missed cleavages were 3; Charge range was positive 2, 3, 4, & 5; Masses were monoisotopic; Precursor Tol was 20 ppm; Frag Tol was 30 ppm; Instrument was ESI Q high res; Constant Mods was none; Variable Mods was oxidation of methionine; User defined modifications were 1) malathion aged, (specificity serine, elemental composition PSO2CH3, accurate mass 109.95 Da, mass modification range 109.5 to 110.5 Da); 2) isomalathion aged (specificity serine, elemental composition PO3CH4, accurate mass 93.98 Da, mass modification range 93.5 to 94.5 Da); 3) malathion adduct (specificity serine, elemental composition PSO2C2H5, accurate mass 123.97 Da, mass modification range 123.5 to 124.5 Da); and 4) isomalathion adduct (specificity serine, elemental composition PSO6C9H15, accurate mass 282.03 Da, mass modification range 281.5 to 282.5 Da). Candidates for peptides with serine adducts were chosen by Protein Prospector and were checked by manual evaluation. Accurate monoisotopic masses were calculated from the elemental composition using Scientific Instrument Services Exact Mass Calculator, single isotope version from Adaptas Solutions (Palmer, MA).

### Hydrolysis of malathion by various esterases

Hydrolysis of the ethyl ester bonds in malathion by recombinant human carboxylesterase, pure human plasma butyrylcholinesterase (from Cohn fraction), and recombinant human acetylcholinesterase was followed with time, using an end-point method as described below. The HuBChE reaction consisted of 1.68 mM malathion and 75 unit/ml (1.76 µM) HuBChE in 1 ml of 20 mM ammonium bicarbonate, in a 15 ml Sarstedt polypropylene tube. The carboxylesterase reaction consisted of 0.27 mM malathion, 0.027 unit/ml rHuCE1 in 1 ml of 20 mM ammonium bicarbonate. The rHuAChE reaction consisted of 0.27 mM malathion, 3.6 unit/ml rHuAChE, in 1 ml 20 mM ammonium bicarbonate. The activities of rHuCE1, HuBChE, and rHuAChE were confirmed before the assays. All reactions were incubated at room temperature in the dark for up to 48 hours.

Reactions were stopped at various timed intervals by adding 3 volumes of acetonitrile, followed by centrifugation at 2600xg for 30 minutes to pellet solids. The supernatant was transferred to a 15 ml Pyrex tube, dried in a SpeedVac, redissolved in 700 µl of 20 mM ammonium bicarbonate, and passed through an Ultrafree-MC 0.22 µm Durapore centrifugal filter (Millipore, cat# UFC30GV0S). Samples were protected from light because malathion alpha monocarboxylic acid appeared to degrade in light in the presence of acetonitrile. Assays were repeated 3-times and average values were used.

### HPLC separation

Filtered samples were loaded onto a Waters 625 HPLC with a Prodigy 5 µm ODS column, 11 x 4.6 mm (Phenomenex, cat# D3300-EO) using a 500 µl loop and eluted with a 36-minute gradient of 0–60% acetonitrile at 1 ml per minute. Solvent A was water. Solvent B was 100% acetonitrile. Elution of malathion and derivatives was detected by absorbance at 220 nm. Our HPLC procedure was patterned after work by Velkoska-Markovska & Petanovska-Ilievska [34].

Five peaks were detected in the HPLC effluent. Peaks were collected and identified by comparison with the elution of standards: malathion; malathion dicarboxylic acid; isomalathion; malathion beta monocarboxylic acid; and malathion alpha monocarboxylic acid.

### Infusion mass spectral analysis of the hydrolysis products

Corresponding peaks from each time point in the hydrolysis of malathion were combined, dried in a SpeedVac, redissolved in water (to an estimated concentration of about 2 mM), passed through an Ultrafree-MC 0.22 µm Durapore centrifugal filter, acidified with 10% formic acid, and infused into an Orbitrap Fusion Lumos mass spectrometer at 5 µl/min. Mass range was 150–600 Da. Sheath gas was set to 0 arbitrary units, and the auxiliary gas was set to 7 arbitrary units.

## Results and discussion

### Inhibition of HuBChE by an isomalathion contaminant in malathion

No inhibition of HuBChE activity was observed in the presence of malathion at concentrations below 0.43 mM, which is the solubility limit of malathion in water. At higher concentrations, up to 126 mM, inhibition was detected, but because the concentration of solubilized malathion could not exceed 0.43 mM, that inhibition can best be ascribed to trace amounts of contaminants in the malathion preparation.

**Fig 2:**
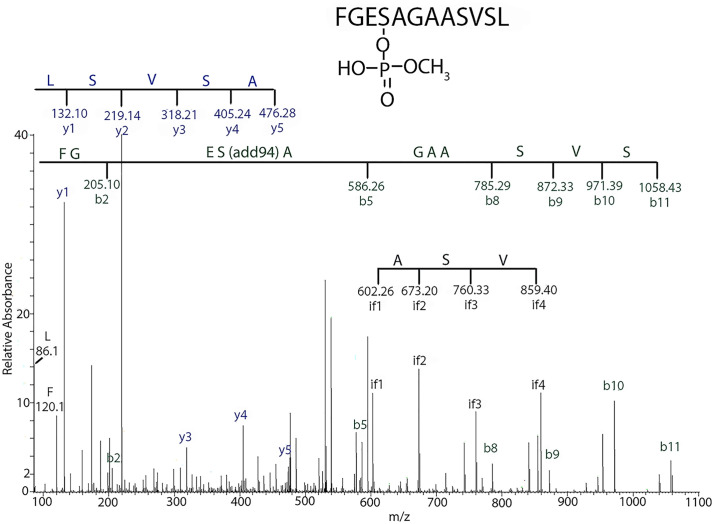
Tandem mass spectral fragmentation spectrum of the active site peptide of HuBChE, accession # P06276, labeled on the active site serine with a 94 Da mass from isomalathion. The blue fragments define a y-ion sequence ASVSL. The green fragments define a b-ion sequence FGESAGAASVSL labeled on the first serine by a 94 Da mass that is consistent with (HO)P(O)OCH_3_, an aged fragment from isomalathion. The black sequence is an internal fragment with sequence ASV. The parent ion has a mass of 595.26 m/z in the charge state +2. The 94 Da added mass on the active site serine of HuBChE is consistent with isomalathion, a contaminant in the malathion preparation. The 94 Da adduct cannot come from malathion. See Fig 3 for theoretical adduct masses for a malathion adduct, none of which were observed.

**Fig 3:**
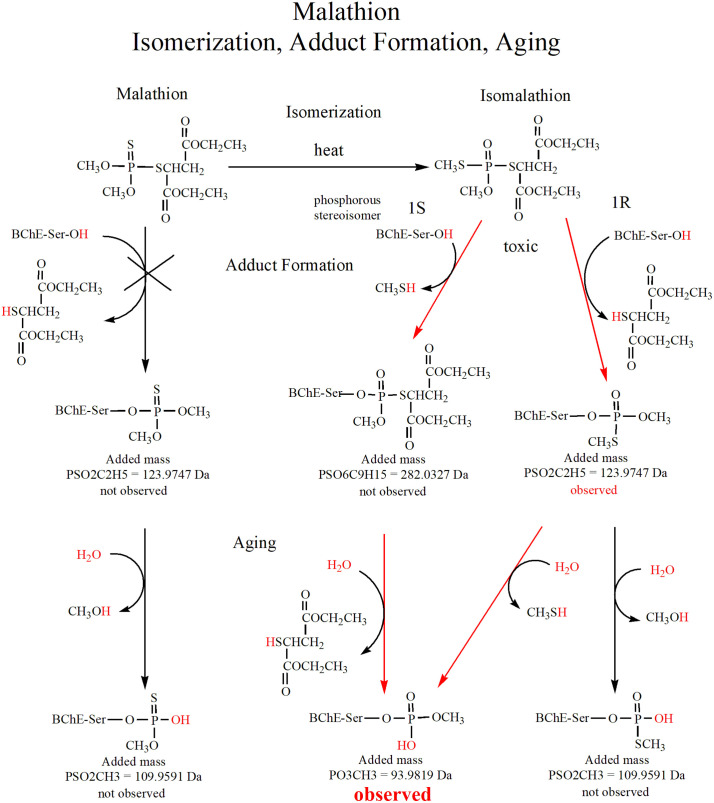
Malathion isomerization, adduct formation, and aging. The red atoms indicate the chemistry at each step. The red arrows highlight the 1S and 1R pathways for formation of the 93.98 Da adduct from isomalathion. The X thru the malathion pathway indicates that species in this pathway are not involved in formation of the 94 Da added mass observed for the adduct from the reaction of HuBChE with malathion. The 94 and 124 Da isomalathion adducts were reported for equine butyrylcholinesterase by Doorn et.al. [36].

**Fig 4:**
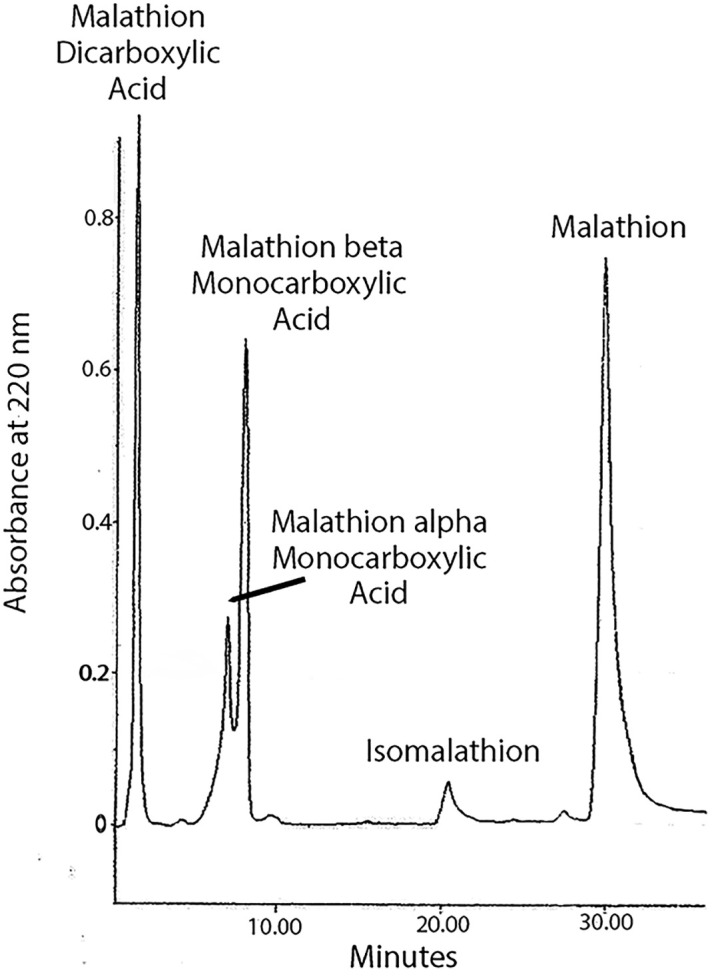
HPLC profile from hydrolysis of malathion by HuBChE. The malathion reaction products were separated on a C18 column which was eluted with a 36-minute gradient of 0–60% acetonitrile at 1 ml per minute, where solvent A was water and solvent B was 100% acetonitrile. Elution of malathion and derivatives was detected by absorbance at 220 nm. The identity of the chemical in each peak was determined by mass spectrometry and by comparison to the elution of standards.

Mass spectral analysis of HuBChE peptic-peptides from the malathion-HuBChE reaction showed a peptide with an adduct on the active site serine of HuBChE with an added mass of 94 Da, Fig 2. This species is consistent with an aged adduct from isomalathion. Fig 3 gives the theoretical adduct masses for species from the reactions of malathion and isomalathion with HuBChE. Potential adducts at 110 and 124 Da from malathion were not detected. The added mass of 94 Da is consistent with an isomalathion adduct. Although the same mass could be formed from malaoxon, it could not come from malathion. That the 94 Da adduct is due to isomalathion and not to malaoxon is demonstrated by HPLC separation of malathion hydrolysis products, Fig 4, which shows a small peak at 21.4 minutes, consistent with isomalathion. Under the same conditions, malaoxon elutes at 19.2 minutes.

Reports from the literature on the reaction of isomalathion with human and horse BChE also describe an adduct with an added mass of 94 Da [35,36]. There are two routes in Fig 3 that lead from isomalathion to the 94 Da adduct. They reflect stereoisomers at the phosphorous of isomalathion. The 1R isomer loses a diethyl thiosuccinate moiety to form a 124 Da adduct, which then loses methanethiol via aging to form the 94 Da adduct. The 1S isomer first loses the methanethiol and then loses the diethyl thiosuccinate [36]. The 1R path is 4-to-7 fold faster than the 1S [37].

### Hydrolysis of the ethyl esters of malathion by various esterases

We explored the hydrolysis of malathion by pure recombinant human carboxylesterase, pure plasma-derived human butyrylcholinesterase, and pure recombinant human acetylcholinesterase in 20 mM ammonium carbonate buffer at room temperature. HPLC separated the hydrolysis mixture into five fractions, Fig 4. These fractions were identified as the starting malathion (eluting at 29.4 minutes), malathion alpha monocarboxylic acid (eluting at 7.0 minutes), malathion beta monocarboxylic acid (eluting at 8.0 minutes), malathion dicarboxylic acid (eluting at 1.3 minutes), and isomalathion (eluting at 20.3 minutes), by comparison to pure samples.

Additional confirmation of the identity of the HPLC peaks in Fig 4 was obtained by mass spectral infusion analysis of each HPLC peak. The 29.4-minute fraction contained a prominent mass of 331.00 Da, consistent with +1 mass of malathion. The 20.3-minute fraction also contained a mass of 331.00 Da. The masses of malathion and isomalathion are identical, but this mass is most consistent with isomalathion because elution of isomalathion occurs at 20.3 minutes. The 8.0-minute fraction contained a mass of 303.00 Da consistent with malathion beta monocarboxylic acid. The 7.0-minute fraction also contained a mass at 303.00 Da consistent with malathion alpha monocarboxylic acid. The 1.3-minute fraction contained a mass of 275.10 Da consistent with malathion dicarboxylic acid.

The malathion peak decreased with time of incubation with HuBChE and rHuCE1, but not with rHuAChE. The malathion alpha monocarboxylic acid, malathion beta monocarboxylic acid and malathion dicarboxylic acid peaks increased with time. The isomalathion peak remained constant. These observations indicate that both ethyl esters of malathion are hydrolyzed by HuBChE and rHuCE1 into the products shown in Fig 4. The ethyl esters of malathion were not hydrolyzed by rHuAChE.

The observed half-lives (t1/2) for hydrolysis of the ethyl esters of malathion by HuBChE and rHuCE1 under first-order conditions were 48 hours (2880 minutes) and 5 minutes, respectively (Table 1). Half-life values have traditionally proven to be extremely valuable when comparing the reaction of a compound with various enzymes [38]. If the enzyme concentrations are of the same order and much less than the reactant concentration, there is an inverse proportionality between t1/2 and the bimolecular rate constant ki (t1/2 = Ln2/ki). Under these conditions, the t1/2 values are a direct reflection of the relative reactivities of the enzymes, i.e., if the t1/2 for enzyme 1 is 10x smaller than the t1/2 for enzyme 2, then enzyme 1 is 10x more reactive than enzyme 2. In our case, the difference in reactivity of the enzymes is so large (cf. observed t1/2 in Table 1) that a wide range of enzyme concentrations had to be used. Comparison of the relative reactivities requires normalization. Multiplying the t1/2 by the amount of enzyme (in moles) will normalize the relative reactivities. For example, for a given enzyme, doubling the amount of enzyme in the reaction will double the rate, which is equivalent to halving the observed t1/2 value. Multiplying the t1/2 value by the moles of enzyme in each reaction will yield comparable rates. When the enzymes are different, the moles of enzyme in the reaction can be obtained by 1) dividing the amount of enzyme in the reaction (units/ml) by the specific activity of the enzyme (units/mg) to obtain mg/ml enzyme, equal to g/L; 2) dividing the g/L by the molecular weight (g/mole) of the enzyme to obtain moles/L; 3) multiplying moles/L by the reaction volume in liters to obtain moles of enzyme in the reaction. Normalization is obtained by multiplying the observed t1/2 by the moles of enzyme in the reaction. The smaller the “t1/2 x moles” number the faster the hydrolysis reaction. These calculations are summarized in Table 1. Hydrolysis of the ethyl ester of malathion by rHuCE1 is 100,000-fold faster (t1/2 x moles = 4x10^^-11^^ min) than hydrolysis by HuBChE (t1/2 x moles = 5.1x10^^-6^^ min).

**Table 1:** Normalization of the hydrolysis of the ethyl esters of malathion t1/2 values.

Enzyme	Reaction Concen units/ml	Specific Activity units/mg	Molecular Weight gm/mole	Reaction Volume ml	Enzyme in reaction moles	Observed t1/2 ± SD minutes	t1/2 x moles of enzyme
rHuCE1	0.027	60^a^	59,000^d^	1.05	8.0x10^-12^	6.3 ± 2.4	5.0x10^-11^
rHuAChE	3.6	6000^b^	65,000^b^	1.10	1.0x10^-11^	0	0
HuBChE	75	500^c^	85,000^f^	1.00	1.7x10^-9^	2880	5.1x10^-6^

aCarboxylesterase specific activity determined with p-nitrophenyl acetate [40]

brHuAChE specific activity determined with acetylthiocholine [41] performed only once

cHuBChE specific activity determined with butyrylthiocholine [42] performed only once

dCarboxylesterase monomer molecular weight [40]

erHuAChE monomer molecular weight [41]

fHuBChE monomer molecular weight [43]

Malathion hydrolyzes spontaneously in water. The half-life at pH 8.16 is 1.65 days [39]. However, hydrolysis is negligible in 20 mM ammonium bicarbonate over 24 hours.

Pure HuBChE hydrolyzed malathion into the two monocarboxylic acids, a result that expands the finding of Main et al [12] who noted hydrolysis of malathion ethyl esters by human carboxylesterase, but not by human blood, where the plasma contains HuBChE and the red cells contain HuAChE.

## Conclusion

Organophosphate pesticides are not all alike. Malathion is an exceptionally safe organophosphorus pesticide. Low dose environmental exposures to malathion do not cause symptoms of acute organophosphate poisoning. OSHA, in its Occupational Chemical Database on malathion (updated 01/28/2021) gives a permissible exposure level of 15 mg/m^^3^^ for a time weighted average over an 8-hour workday and a 250 mg/m^^3^^ exposure level as being immediately dangerous to life or health.

Toxic organophosphate pesticides inhibit acetylcholinesterase. The consequences of inhibiting acetylcholinesterase are a loss of nerve impulse transmission that ultimately causes muscle paralysis, inhibition of breathing, and death [44].

**Fig 5:**
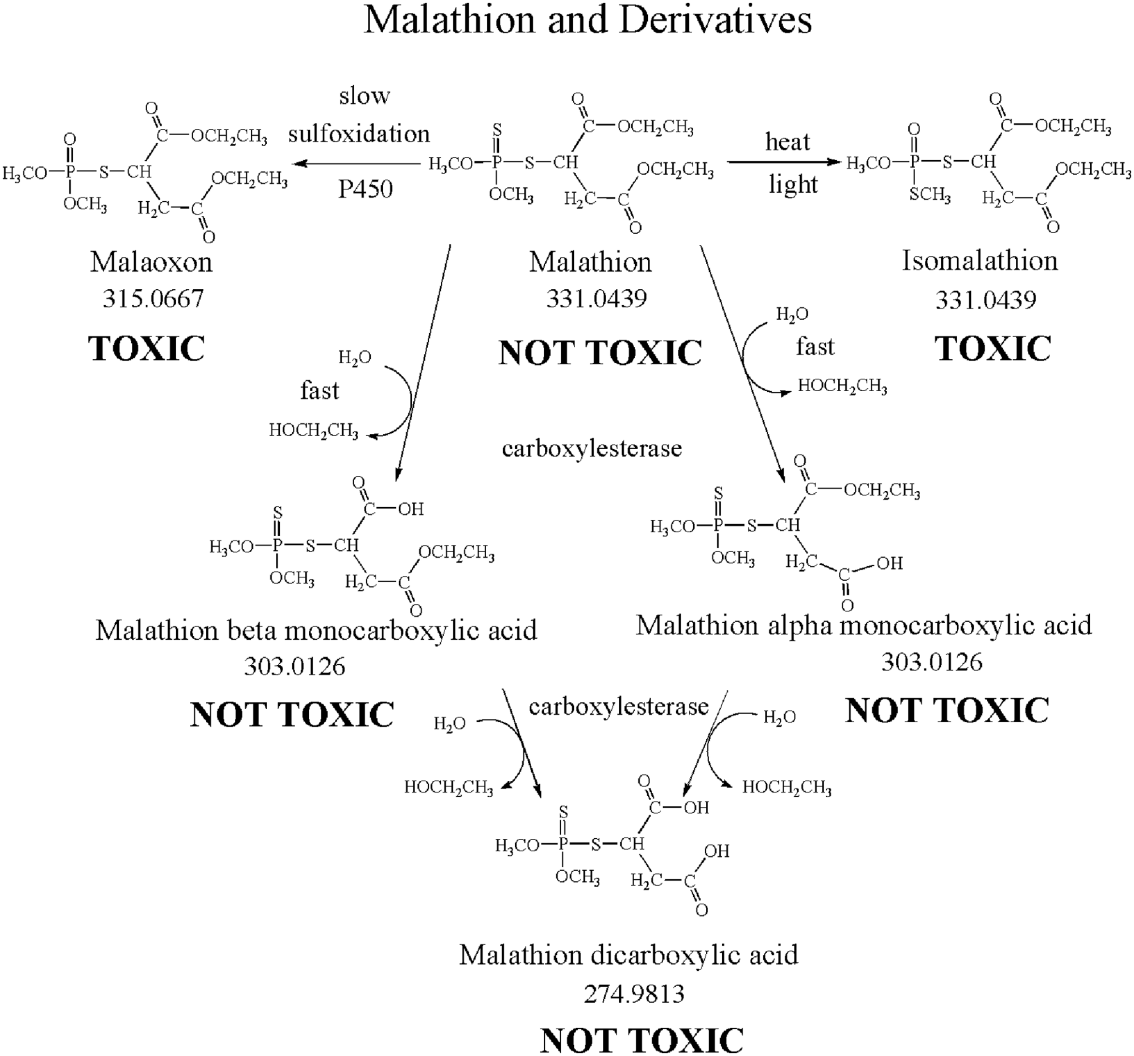
The malathion metabolite, malaoxon, and the degradation product, isomalathion, are toxic. Malaoxon and isomalathion inhibit HuAChE, HuBChE, and human carboxylesterase. The de-esterified malathion monocarboxylic and dicarboxylic acids are not toxic. They do not inhibit HuAChE, HuBChE, or human carboxylesterase. Our findings suggest that malathion is safe at low environmental exposures because the rate of formation of the nontoxic metabolites is faster than the rate of formation of the toxic derivatives.

The mechanism to explain malathion safety is summarized in Fig 5. Unlike other agricultural pesticides malathion does not inhibit acetylcholinesterase, butyrylcholinesterase, or carboxylesterase. The toxic form of malathion is malaoxon. Malaoxon binds to and inactivates acetylcholinesterase, butyrylcholinesterase and carboxylesterase. However, the esterases in our lungs, liver, kidney and intestines prevent malaoxon from being formed when the malathion dose is low. The esterases, in particular carboxylesterase, detoxify malathion by converting malathion to its acids. The acid forms of malathion and malaoxon are not inhibitors of human acetylcholinesterase. The mechanism to explain the low mammalian toxicity of malathion has been convincingly established by others [11,13,45], but is often overlooked. The present report is a reminder that malathion has built-in safety features that are absent in other organophosphorus pesticides.

Comparison of accepted ambient exposure thresholds for malathion, i.e., the OSHA permissible exposure limit (PEL) and the EPA population adjusted reference dose (PAD-RfD) with the observed carboxylesterase hydrolysis parameters is informative. The OSHA PEL is 15 mg malathion per cubic meter, which means that it is safe for a person to work for 8 hours in a space that contains 15 mg malathion/m^^3^^ of air [46]. The PEL RfD is 0.14 mg/kg/day/person, which means that it is safe for a person to ingest 0.14 mg malathion per kilogram body mass per day [47].

The OSHA and EPA malathion exposure levels are lower than the amount of carboxylesterase 1 in human liver, see Table 2 and associated calculations in the table footnotes for details. From this, one would expect that very little malathion would escape the liver. In addition, the half-life for hydrolysis of malathion by carboxylesterase in the liver is remarkably short (0.009 minute) which means that malathion is detoxified very rapidly. Both of these factors re-enforce the proposal that malathion is not toxic to humans. While our findings support a lack of direct toxicity from malathion at low exposures, broader health outcomes require continued surveillance.

**Table 2:** Comparison of ambient threshold values for malathion with carboxylesterase hydrolysis rate.

OSHA PEL µmoles/liter air/day ^A^	EPA RfD µmoles/day ^B^	Carboxylesterase 1 t1/2 in liver microsomes minutes ^C^	Carboxylesterase 1 in liver microsomes µmoles ^C^
0.045	30	9x10^-3^	76

A OSHA PEL conversion to moles malathion

15 mg/m^3^/day x m^3^/1000 L = 15x10^-2^ mg/L/day x 10^−3^ gm/mg = 1.5x10^-5^ gm/L/day

1.5x10^-5^ gm/L/day ÷ 330 gm/mole (MW of malathion) = 4.5x10^-8^ mole/L/day

B EPA RfD conversion to moles malathion.

Assume a 70 kg adult.

0.14 mg/kg/day x 70 kg = 9.8 mg/day x 10^-3^ gm/mg = 9.8x10^-3^ gm/day

9.8x10^-3^ gm/day ÷ 330 gm/mole (MW of malathion) = 3.0x10^-5^ mole/day

C Calculation of carboxylesterase 1 concentration in human liver microsomes

Assume that malathion is hydrolyzed in the liver by microsomal carboxylesterase 1 (CE).

Adult human liver microsomes contain 1600 pmole CE/mg total liver protein [48].

1600 pmole CE/mg microsomal protein = 1.6x10^-9^ mole CE/mg microsomal protein

1.6x10^-9^ mole CE/mg microsomal protein ÷ 10^-3^ gm/mg = 1.6x10^-6^ mole CE/gm microsomal protein

Adult human liver contains 32 mg microsomal protein/gm liver [49].

32 mg microsomal protein/gm liver x 10^-3^ gm/mg = 3.2x10^-2^ gm microsomal protein/gm liver

3.2x10^-2^ gm microsomal protein/gm liver x 1.5x10^3^ gm liver = 48 gm microsomal protein/liver

48 gm microsomal protein/liver x 1.6x10^-6^ mole CE/gm microsomal protein = 7.6x10^-5^ mole CE/liver

Determination of t1/2 for the reaction of CE in liver by comparison of the molar concentration of CE in liver to molar concentration of CE in the assay where the t1/2 value is directly proportional to the CE concentration.

8.0x10^-12^ moles CE in reaction ÷ 1.05x10^-3^ L = 7.6x10^-9^ M CE in assay yielded a t1/2 = 6.3 minutes (see Table 1)

7.6x10^-5^ mole CE/liver ÷ 1.5 L/liver = 5.1x10^-5^ M CE in liver

6.3 minutes x 7.6x10^-9^ M CE in assay ÷ 5.1x10^-5^ M CE in liver = 9x10^-3^ min for t1/2 in liver

High doses of malathion are toxic. Drinking a malathion solution or spilling concentrated malathion on skin results in symptoms of cholinergic toxicity. High doses of malathion include toxic levels of isomalathion and other contaminants. Contaminating levels of isomalathion have been associated with poisoning due to malathion. For example, in 1978, an epidemic of malathion poisoning was correlated with the level of isomalathion in commercial malathion preparations [50]. In addition, isomalathion was isolated from technical malathion preparations and shown to increase upon storage for 3–6 months at 40°C [51].Isomalathion inhibits the esterases thereby blocking their detoxifying role. Isomalathion inhibition of carboxylesterase is especially dangerous, because carboxylesterase has a major role in detoxifying malathion and malaoxon. In the absence of the detoxifying activity of carboxylesterase, malaoxon accumulates to levels that inhibit acetylcholinesterase. Toxic signs appear only after more than 50% of acetylcholinesterase activity is inhibited in muscle and brain. It is noteworthy to point out that our laboratory-based results do not provide direct evidence on the toxicity of low dose exposure to malathion. On the other hand, epidemiological studies yield correlations between malathion exposure and toxicity, but do not constitute cause-and-effect relationships.

There is no convincing evidence for an association between low dose malathion exposure and cancer. Animal and cultured cell studies showing potential cancer risks from exposure to malathion employed supra-environmental doses. The International Agency for Research on Cancer says that malathion is not classifiable as to its carcinogenicity in humans.

Possible long term or subacute toxic effects cannot be ruled out.
